# Effects of worm control practices examined by a combined faecal egg count and questionnaire survey on horse farms in Germany, Italy and the UK

**DOI:** 10.1186/1756-3305-2-S2-S3

**Published:** 2009-09-25

**Authors:** Georg von Samson-Himmelstjerna, Donato Traversa, Janina Demeler, Karl Rohn, Piermarino Milillo, Sandra Schurmann, Riccardo Lia, Stefania Perrucci, Antonio Frangipane di Regalbono, Paola Beraldo, Helen Barnes, Rami Cobb, Albert Boeckh

**Affiliations:** 1 University of Veterinary Medicine, Hannover, Germany; 2 Faculty of Veterinary Medicine, University of Teramo, Italy; 3 Faculty of Veterinary Medicine, University of Bari, Italy; 4 Faculty of Veterinary Medicine, University of Pisa, Italy; 5 Faculty of Veterinary Medicine, University of Padua, Italy; 6 Faculty of Veterinary Medicine, University of Udine, Italy; 7 Fort Dodge Animal Health, Southampton, UK; 8 Fort Dodge Animal Health, Princeton, USA

## Abstract

**Background:**

For the control of worm infections, the strategic use of anthelmintics, often accompanied by additional farm and/or pasture management procedures, is currently applied on most horse farms in industrialized countries. However, the particular effects of the specific worm control procedures are often unclear and have only been investigated to a limited extent. We examined faecal egg count (FEC), faecal egg count reduction (FECR) and questionnaire data on farm and pasture management procedures. The aim of this study was to determine whether specific worm control practices reported to be applied in European horse farms affect worm prevalence.

**Results:**

This study involved 20 German, 26 Italian and 16 UK horse farms for each of which FEC were performed on a minimum of 16 horses. In total, 2029 horse faecal samples were quantitatively analysed for helminth eggs, resulting in 56.3% of the faecal samples being positive for strongylid eggs. The prevalence in the 742 German horse samples (48.1%) was significantly lower than that in the 737 Italian (61.1%) and the 550 UK (60.9%) samples. As expected, a significant effect of horse age on the infection prevalence was observed, with adult horses showing lower prevalences and lower mean FEC than foals and yearlings. The majority of the participating farms were stud farms (n = 29), followed by riding stables (n = 27) and racehorse stables (n = 6). The prevalence of strongyle infection by farm type differed between countries. While in Germany, horses on riding farms were significantly less often strongyle positive, in the UK horses on stud farms showed the lowest strongyle prevalences, whereas in Italy, no significant difference between farm types were seen. On all farms, horses received routine/preventive anthelmintic treatment. An effect of treatment frequency on strongyle prevalence was only encountered with adult horses. On farms performing more than one annual treatment, faecal samples were significantly less often positive. Furthermore, by comparing the FECR results of individual horses with their pre-treatment FEC, it was found that high pre-treatment FEC were associated with a significantly higher probability for a FECR below 90%.

**Conclusion:**

Overall, age-dependent strongyle infection patterns and general worm control approaches were found to be similar on horse farms in the three countries. Also, a negative association of pre-treatment FEC and treatment efficacy was consistently found in all countries. However, mean strongyle prevalences and frequencies of anthelmintic treatments were considerably different. In addition to the age-dependent prevalence patterns, the finding of a possible negative association between high FEC and reduced FECR might argue for a focus on horses showing high pre-treatment FEC when monitoring anthelmintic treatment efficacy in the field.

## Background

To date, worm control in equines is in most cases based on the exclusive and regular use of anthelmintic drugs. However, due to the increasing spread of anthelmintic resistance (AR) this approach has to be considered unsustainable. The prevalence of resistance in cyathostomins against benzimidazole (BZ) type drugs is increasing [1]. This means that the horse industry mainly has to rely on products from two drug classes, i.e. the macrocyclic lactones (ML) and the tetrahydropyrimidines. Resistance against pyrantel (PYR), the only important member of the tetrahydropyrimidines for use in horses, has remained fairly low in comparison to the benzimidazole drugs in most countries, based on current criteria of resistance. Nevertheless, PYR resistance occurs and has been described in a number of European countries such as Denmark, Italy and Sweden [2-4], but not for Germany or the UK. The ML drugs, the most commonly administered anthelmintics in horses, have apparently retained their high efficacy against cyathostomins despite over 20 years of use. However, due to the increasing reliance on ML, most experts in equine parasitology suspect that ivermectin (IVM) resistance in cyathostomins is inevitable [1]. Recently, a large study on a total of 102 horse farms in Germany, Italy and the UK found indications for IVM resistance in cyathostomins on one Italian and two UK yards [5]. Additionally, for the first time, PRY resistance in Germany and UK was found as well as an indication for triple-resistance, i.e. against fenbendazole (a BZ), PYR and IVM, was encountered in cyathostomins on one yard in the UK.

Key factors contributing to the development of AR are high treatment frequencies, prolonged use of the same drug class, high stocking rates, under-dosing and the off-label use of anthelmintic drugs [6-9]. In some countries, a further problem of equine parasite control is the decreasing involvement of veterinarians in therapy [10,11]. Instead, worm control is often decided and carried out by horse owners and stable managers. Denmark has responded to this problem by making anthelmintic drugs available only by prescription and prohibiting their use for routine, prophylactic treatment [12].

Initially identified as an important factor for development of AR in sheep nematodes, the size of the parasite refugium is now also considered to be of relevance in horse parasites [12,13]. Refugium describes the proportion of a parasite population that is not exposed to the drug at the time of treatment. For example, the free-living stages on pasture constitute a major part of the refugium, but also parasites in untreated individuals are in refugium. Additionally, parasitic stages which do not come into contact with the drug are considered to be in refugium [14]. Since parasites in refugia are not under selection pressure for AR, they provide a source of susceptible alleles in the population [13,15]. The maintenance of an adequate proportion of the total parasite population in refugium can slow down the development of AR as has been confirmed by experimental studies with sheep [16,17] as well as by computer modelling [18,19].

As outlined above, AR in horse nematodes is a further evolving phenomenon. Thus, current worm control strategies need to take the consequences of spreading AR into account and should be evaluated for their effects on development of resistance in addition to the maintenance of horse health. Improved control strategies may lead to a more sustainable use of anthelmintics in horses.

The aim of the present study was to evaluate the current worm control strategies in horses employed in three European countries, and to investigate if specific farm or pasture management characteristics are associated with different levels of worm prevalence. Finally, such information is expected to be helpful for optimizing the interaction of chemical and non-chemical worm control measures.

## Materials and methods

A combined faecal egg count (FEC) and questionnaire survey was performed during summer of 2008 on 20 German, 26 Italian and 16 UK horse farms with a history of no anthelmintic treatment in the past 12 weeks. Per farm, if possible all (i.e. never less than 50% of the total number of horses on farm) but at least 16 horses were coproscopically examined using a modified McMaster method with a sensitivity of at least 50 egg per gram [5]. For each farm, a questionnaire consisting of 23 questions about farm indicators such as farm type, number of animals per farm, age composition of horse stock; pasture management including duration of access to pasture, size of available pasture, removal of faeces, fertilization and treatment regimes (e.g. frequency of treatment, quarantine treatments, weight assessment procedures) was completed. Farms were categorized into the following three types: riding stable (FT1), stud farm (FT2) and racehorse stable (FT3) according to the total number of horses, annual number of newborn foals and type of use.

The collected data were prepared for the present work in such a manner, that "horse" can be used as statistical unit in regression analytical calculations. Besides taking into account age as a covariable in regression models, horses were also grouped into age classes with foals (up to one year old), yearlings (over one year up to three years old) and adults (over three years old) for further statistical analyses. Following dichotomization of the dependent variables (FEC for strongyles) in "non-infected" if FEC was zero and "infected" otherwise, logistic regression models were calculated. Multiple logistic regression was used for assessing association between predictors (risk factor like farm type or horse age) and a binary outcome predicting the probability for presence of infection, statistically adjusted for potential confounding effects of other covariates. A fraction (up to 20 horses per farm) of the examined horse population also participated in an anthelmintic efficacy trial [5]. In the present study the respective pre-treatment FEC data were included. The individual horse FECR percentages were dichotomized to "resistant" if FECR is less than 90% and "sensitive" if FECR is equal or greater than 90%. Logistic regression was used for calculating a putative association of the dichotomized FECR with the respective pre-treatment FEC. Wald's odds ratios including confidence intervals were calculated and partly diagrammed. Analyses were carried out with the statistical software SAS, Version 9.2 (SAS Institute, Cary, NC, USA). The generalized linear models were calculated with the procedure "LOGISTIC", furthermore the SAS Procedure "FREQ" was used for calculating frequencies of prevalence depending on independent variables in cross-tabulations. Differences in FEC between countries within age groups were analysed using contingency tables with chi-square-statistic. Error probability less than five percent (p < 0.05) were considered statistically significant.

## Results

**Figure 1:**
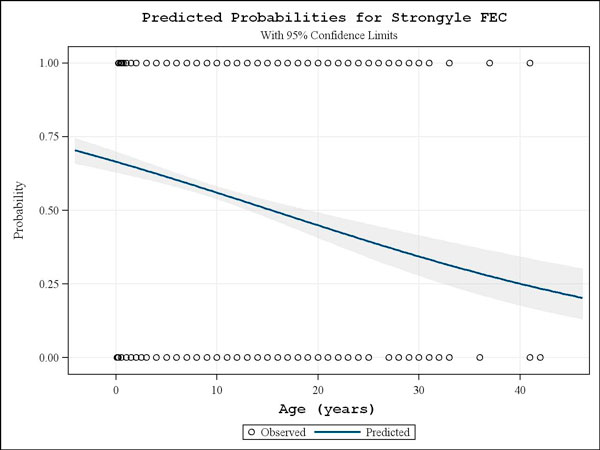
**Predicted probabilities for strongyle FEC**. Probability of strongyle infection controlled by age, showing the influence of horse-age on prevalence for the total 2029 horse samples examined. On the y-axis the predicted probabilities for the FEC status following dichotomization as strongyle negative (0) or positive (1) is depicted. The 95% confidence interval is given as shaded area.

In total, data from 62 questionnaires and FEC results from 742 German, 737 Italian and 550 UK horses were analysed in this study. The majority of horses examined were adults (529, 582 and 470 for Germany, Italy and the UK, respectively), followed by yearlings (127/129/18) and foals (86/26/62). A strong effect of age on the strongyle infection rate (Fig. 1) was encountered for the complete data set showing significantly reduced infection probabilities with increasing age (p < 0.001). When comparing the infection rates in the three countries, it was found out that the strongyle prevalence was significantly higher (p < 0.001) in the participating Italian and UK farms, 61.1% (95% confidence interval (CI): 57.4-64.6) and 60.9% (95% CI: 56.7-65.0), respectively, than that observed in the German farms which was 48.1% (95% CI: 44.5-51.7).

**Figure 2:**
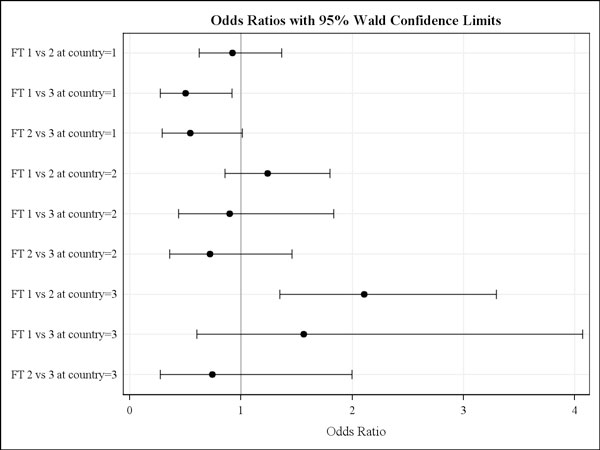
**Strongyle infection risk between farm types for each country**. Odds ratios with 95% Wald confidence limits for strongyle infections risk between farm types (FT, i.e. 1 = riding stable, 2 = stud farm, 3 = racehorse stable) for the three countries involved (i.e. 1 = Germany, 2 = Italy, 3 = UK).

The selection of farms was done based on the number of horses available for sampling and the fulfilment of the other study inclusion criteria (e.g. no recent treatment, willingness to participate) but not based on the individual farm type. According to the questionnaire data, in Germany, Italy and the UK, eight, ten and nine riding stables, nine, fourteen, and six stud farms and three, two and one race horse farms were included, respectively. When examining the effect of farm type using odds ratio calculations stratified according to country, two statistically significant observations were made only for adult horses. For the German farms horses, riding stables had a 0.5 (95% Wald confidence limits: 0.27-0.92) infection risk compared with that in racehorse stables. In the UK, riding stables were associated with a 2.1 times higher estimated infection risk (95% Wald confidence limits: 1.35-3.30) compared with those on stud farms (Fig. 2).

Various farm and pasture management procedures such as removal of faeces, fertilising and frequency of anthelmintics treatment were assessed for their potential effect on strongyle prevalences. Routine (i.e. at least once per month) removal of faeces from pasture was performed on three German, ten Italian and four UK farms. Based on the statistical analysis of the present data, this procedure was not found to be consistently associated with reduced strongyle infection rates. Also no clear effect on strongyle prevalence was obtained from any of the other examined pasture management practices.

**Figure 3:**
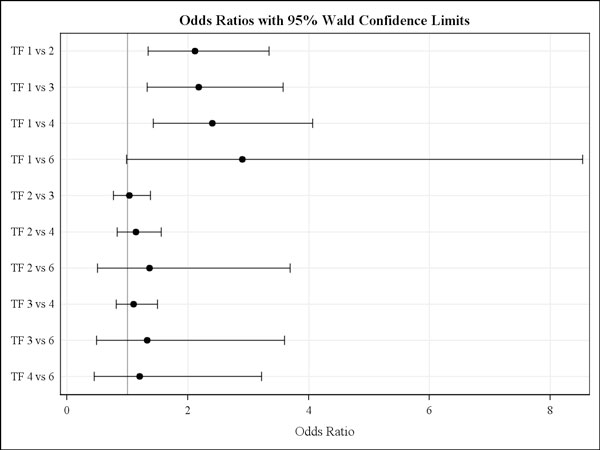
**Strongyle infection risk by reported treatment frequencies for adult horses**. Odds ratios with 95% Wald confidence limits for strongyle infection risk in adults by anthelmintic treatment frequency (TF) ranging from 1 to 6 annual treatments. The data were analysed following stratification for age group, where only for adults a significant influence of treatment frequency was observed.

Preventive or routine anthelmintic treatments were recorded for all participating farms. There was a clear difference in the reported treatment frequencies between age classes and countries. The average number of annual treatments reported to be given on German farms to foals was 6.3, to yearlings 3.7 and to adults 3.0 treatments. In contrast, foals on Italian farms received fewer anthelmintic treatments per year (i.e. 2.3) and no clear difference between age groups in treatment frequency was observed. Mean annual treatment frequencies in foal, yearlings and adults recorded on UK farms were 5.0, 4.5 and 3.2, respectively. There appeared to be an effect of anthelmintic treatment frequency on the mean strongyle prevalence in horses stratified by age group. However, this was only seen in adult horses, where those receiving only one compared with those receiving two to four yearly treatments were found to have an approximately two to four fold higher risk to be strongyle FEC positive according to odds ratio calculations (Fig. 3). Noteworthy, for more frequent treatments the present data did not show a significant effect concerning the reduction of strongyle infection risk. The most often (54 of the farms) used method for the assessment of bodyweight for dose calculation was visual assessment. Three UK and one Italian farm used a girth tape to evaluate the bodyweight while the remaining four farms employed a balance.

**Figure 4:**
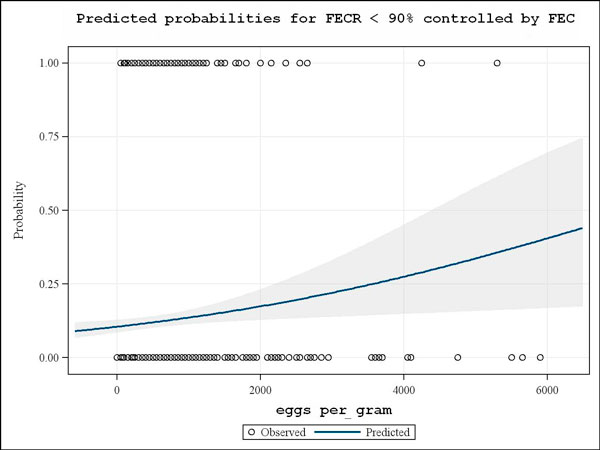
**Predicted probabilities for FECR <90% controlled by FEC**. Logistic regression analysis showing increasing predicted probabilities for FECR <90% (without differentiation of drug class used for treatment) with higher stronglye egg per gram counts. On the y-axis the FECR result following dichotomization according to 0 for FECR ≥ 90% and 1 for FECR<90% is depicted. The 95% confidence interval is given as shaded area.

The FEC of those horses furthermore involved in a FECRT trial were comparatively analysed with the respective FECR results. When examining pre-treatment egg-per-gram counts towards a potential effect on the individual horse FECR a significant (p < 0.001) negative association was recorded. Higher pre-treatment FEC were found to correspond with a higher predicted probability for a FECR below 90% (Fig. 4).

## Discussion

In the present study, results from a questionnaire survey performed in combination with faecal analysis of representative numbers of horses from German, Italian and UK farms were comparatively analysed. Possible effects of pasture management, pasture and stable hygiene, farm management, treatment regime and assessment of weight on the prevalence of strongyle infections were particularly investigated.

The mean strongyle prevalence in the countries observed herein was similar to findings of previous studies. While on German horse farms, prevalences of approximately 40-60% were reported [20,21], on Italian farms, prevalences between 80-100% were previously described [22,23]. Furthermore, the findings confirm that young horses show the highest incidence of patent strongyle infections, which is presumably associated with less developed age-dependent immunity. This can lead to an increased infection pressure due to a more heavily contaminated environment of young horses and result in a higher risk of re-infection and shorter prepatent period [24,25].

Pasture hygiene procedures such as regular removal of faeces has previously been recommended as an effective worm control approach resulting in reduced strongyle infection rates in horses [26]. In the present investigation, horses on farms using this practice did not show lower strongyle FEC prevalences. However, it should be noted that due to the limited number of farms performing pasture cleaning, possible confounding factors such as stocking density or the age composition could not be excluded by the statistical examination. Generally, it may be expected that depending on weather conditions removal of faeces at least once or twice weekly is required to achieve an effect on worm burdens.

In general, horses on Italian farms were treated less frequently with anthelmintics than those on German or UK farms. A differentiated management programme for different age groups was suggested as being a useful method providing adequate control [8] because young horses require more frequent treatment than adult horses [24,27,28]. As also observed within a recent similar study on German horse farms [29], the strongyle prevalence was lower in adults treated more frequently, while such an effect was not consistently found for the other two age groups. Based on the present German questionnaire data, foals and yearlings were treated more often than adults. On average, foals received more than 6 treatments during their first year. This was particularly the case for foals on stud farms where approximately one third was treated on a monthly basis (data not shown). Such an intensive treatment frequency should be a matter of concern, since a direct relationship has been shown between the frequency of treatment and the rate of AR development [6,26,30,31]. Additionally, an over-protective dosing strategy, while highly effective in the short term, will probably not be sustainable due to increasing development of AR [8]. Selection pressure for AR will be increased by treatment intervals as long as or even shorter than the prepatent period or the respective egg reappearance period [32]. Susceptible worm populations will not reach patency in the short period between treatments. This results in the next parasite generation mainly represented by resistant individuals. In addition, development of acquired immunity in young horses may be compromised by frequent dosing strategies [24,31,33,34].

Newly introduced horses should be treated with an effective, ideally larvicidal, anthelmintic drug or a combination of different anthelmintic drugs at arrival since they can introduce AR in a herd by harbouring anthelmintic resistant parasites [8,10]. It is also recommended to evaluate the success of any quarantine treatment to avoid introduction of resistant populations [11]. According to the present questionnaire survey, on about 30% of the farms all new arrivals receive anthelmintic treatment, but in no case was the efficacy checked post-treatment (data not shown). On the vast majority of the participating farms the dose calculation was done based on visual assessment of horse weight. This will often lead to underdosing which in turn can propagate anthelmintic resistance [9,32]. It should therefore be recommended to use more precise means of weight assessment like the use of a girth tape where scales are unavailable.

The 90% FECR threshold used when studying a potential effect of FEC on anthelmintic efficacy was chosen according to the respective guidelines for the detection of anthelmintic resistance in horses by the World Association for the Advancement for Veterinary Parasitology [35]. This guideline specifically refers to the use of BZ. However, since other drug classes are not excluded nor further guidelines referring to those the same threshold for all three drug classes were applied. This was done not withstanding that particularly the ML drugs have a higher intrinsic efficacy so that early resistance detection may be problematic with this threshold and thus future guidelines may suggest drug class-specific thresholds. The finding of a potential trend for lower anthelmintic efficacies in individual horses shedding higher numbers of strongyle eggs could be of significance for the optimization of treatment. Consideration should be given to preferentially performing post-treatment FEC in such high egg shedders as means of monitoring anthelmintic efficacy. The reasons for such an effect remain unclear at present. Anthelmintic treatment efficacies are generally considered to be suboptimal in diseased or not fully immunocompetent animals. Accordingly, it may be speculated that horses failing to effectively control their worm burden may also posses a less well developed capacity to support the effect of anthelmintic treatment, i.e. to eliminate worms only partially affected by treatment.

## Conclusion

Comparing strongyle infection patterns, an age-dependent distribution was found for horses in all three countries, however, with clear quantitative differences as demonstrated by significantly higher prevalences in Italian and UK horses. General worm control approaches were found to be similar on German, Italian and UK horse farms. However, mean frequencies of anthelmintic treatments were considerably different, particularly concerning foals on German and UK farms, where significantly more treatments were applied compared with those on Italian farms. Due to the similarity concerning the situation in Germany and the UK it appears questionable that this difference in anthelmintic treatment frequency in foals contributed to the lower overall strongyle prevalence in German horse and also no other factors were identified in this respect. A beneficial effect of anthelmintic treatment demonstrated by significantly lower strongyle infection probabilities was encountered in adult horses when treated at least twice per year. Interestingly, the present data suggest that horses with higher FEC, when treated with anthelmintics, will generally have a higher likelihood of showing a reduced FECR. Accordingly, when resources for worm control monitoring are limited, it seems advisable to focus post-treatment egg counts on younger horses with high FEC.

## List of abbreviations used

AR: anthelmintic resistance; BZ: benzimidazole(s); CI: confidence interval(s); FEC: faecal egg count(s); FECR: faecal egg count reduction; FREQ: frequency; FT: farm type; IVM: ivermectin; ML: macrocyclic lactone anthelmintics; PYR: pyrantel.

## Competing interests

Fort Dodge Animal Health provided financial and logistic support to parts of the study. The authors confirm that this support neither influenced the conceptual design, nor the conduct, the interpretation or any other scientific aspect of the study.

## Authors' contributions

GvSH contributed to the design of the study, was responsible for the German farms study, participated in the statistical analysis and drafted the manuscript; DT contributed to the design of the study, was responsible for the Italian farms study and for the coproscopical analysis of all faecal samples; JD participated in the faecal sampling and the questionnaire survey on German farms, participated in the statistical analysis; KR was responsible for the statistical analysis; PM participated in the faecal sampling and the questionnaire survey on Italian farms, conducted the coproscopical analysis; SS participated in the faecal sampling and the questionnaire survey on German farms; RL participated in the faecal sampling and the questionnaire survey on Italian farms; SP participated in the faecal sampling and the questionnaire survey on Italian farms; AFdR participated in the faecal sampling and the questionnaire survey on Italian farms; PB participated in the faecal sampling and the questionnaire survey on Italian farms; HB contributed to the design of the study, was responsible for the UK farms study; RC co-initiated the study and contributed to the design of the study; AB co-initiated the study and contributed to the design of the study.
